# CD103+ Tissue Resident T-Lymphocytes Accumulate in Lung Metastases and Are Correlated with Poor Prognosis in ccRCC

**DOI:** 10.3390/cancers14061541

**Published:** 2022-03-17

**Authors:** Christine Sanders, Almotasem Salah M. Hamad, Susanna Ng, Racha Hosni, Jörg Ellinger, Niklas Klümper, Manuel Ritter, Carsten Stephan, Klaus Jung, Michael Hölzel, Glen Kristiansen, Stefan Hauser, Marieta I. Toma

**Affiliations:** 1 Institute of Pathology, University Hospital Bonn (UKB), 53127 Bonn, Germany; christine.sanders@ukbonn.de (C.S.); s4alhama@uni-bonn.de (A.S.M.H.); racha.hosni@ukbonn.de (R.H.); glen.kristiansen@ukbonn.de (G.K.); 2 Institute of Experimental Oncology, University Hospital Bonn, University of Bonn, 53127 Bonn, Germany; susanna.ng@ukbonn.de (S.N.); niklas.kluemper@ukbonn.de (N.K.); michael.hoelzel@ukbonn.de (M.H.); 3 Institute of Urology, University Hospital Bonn (UKB), 53127 Bonn, Germany; joerg.ellinger@ukbonn.de (J.E.); mritter@ukbonn.de (M.R.); stefan.hauser@ukbonn.de (S.H.); 4 Department of Urology, Berlin Institute for Urologic Research, Charité-Universitätsmedizin Berlin, CCM, 10117 Berlin, Germany; carsten.stephan@charite.de (C.S.); klaus.jung@charite.de (K.J.)

**Keywords:** ccRCC, immune cell infiltrate, prognosis, CD103+ T-lymphocytes, metastases

## Abstract

**Simple Summary:**

Clear cell renal cell carcinoma (ccRCC) is a highly immunogenic tumor, with highly heterogeneous responses to immune checkpoint inhibition therapy. The role of immune cells infiltrating distant metastases and their impact on prognosis and response to immunotherapy are still unclear. To elucidate the prognostic value of immune cell subtypes, we analyzed the immune cell infiltrate in ccRCC primary tumors and paired distant metastases. We hypothesized that specific subtypes of immune cells may be involved in the control of metastases and may have an impact on the prognosis of ccRCC.

**Abstract:**

Clear cell renal cell carcinoma (ccRCC) is a highly immunogenic tumor with variable responses to immune checkpoint therapy. The significance of the immune cell infiltrate in distant metastases, their association with the immune infiltrate in the primary tumors and their impact on prognosis are poorly described. We hypothesized that specific subtypes of immune cells may be involved in the control of metastases and may have an impact on the prognosis of ccRCC. We analyzed the immune microenvironment in ccRCC primary tumors with distant metastases, paired distant metastases and non-metastasized ccRCC (*n* = 25 each group) by immunohistochemistry. Confirmatory analyses for CD8+ and CD103+ cells were performed in a large ccRCC cohort (*n* = 241) using a TCGA-KIRC data set (ITGAE/CD103). High immune cell infiltration in primary ccRCC tumors was significantly correlated with the development of distant tumor metastasis (*p* < 0.05). A high density of CD103+ cells in ccRCC was more frequent in poorly differentiated tumors (*p* < 0.001). ccRCCs showed high levels of ITGAE/CD103 compared with adjacent non-neoplastic tissue. A higher density of CD103+ cells and a higher ITGAE/CD103 expression were significantly correlated with poor overall survival in ccRCC (log rank *p* < 0.05). Our results show a major prognostic value of the immune pattern, in particular CD103+ cell infiltration in ccRCC, and highlight the importance of the tumor immune microenvironment.

## 1. Introduction

Kidney cancer is the third most common tumor of the urinary tract. The most common histological subtype of renal carcinoma is the clear cell subtype, followed by the papillary and the chromophobe subtypes [1]. About 30% of patients present with an advanced stage of the disease or with distant metastases at the time of diagnosis [2]. ccRCC is known to be an immunogenic tumor, with a high density of infiltrating immune cells. As such, immune checkpoint inhibition has been reported to be an effective therapy towards metastasized ccRCC [3]. However, the association between the composition of immune cell infiltrate and prognosis in ccRCC is still controversial. For example, in contrast to the study published by George et al. [4], a study by Choueiri et al. [5] has reported that high CD8+ T-cell infiltration and PD-L1 positivity in tumor cells is associated with shorter survival in metastatic ccRCC treated with tyrosine kinase inhibitors. A recent study investigating the association of immune cell signatures with the tumor mutational burden (TMB) in ccRCC has shown that tumors with a high TMB had poorer prognosis and a lower signature of CD4+ T-cells, CD8+ T-cells, dendritic cells and M1 and M2 macrophages compared to tumors with a lower TMB [6].

A relapse after tumor resection was associated with lower T-cell infiltration, a lower adaptive immune response and higher neutrophil infiltration [7]. An accumulation of tissue-resident memory CD103+ T-lymphocytes has been associated with a better prognosis in head and neck tumors [8] and lung cancer [9]. In the context of ccRCC, only one study has described a higher infiltration of CD103+ lymphocytes in the peritumoral stroma to be associated with improved prognosis [10]. In contrast to this observation, a recent study showed that CD103+ cancer-specific exosomes in cancer stem cells accumulate in lung metastases and promote the epithelial–mesenchymal transformation of ccRCC [11].

At present, few studies concerning the immune cell infiltrate in distant metastases of ccRCC have been published. Among those, one study has shown a correlation between immune cell infiltration in lung metastases and the infiltration pattern in the primary tumor [12]. In contrast to metastatic colorectal cancer, a high infiltration by DC-LAMP^+^ mature dendritic cells and CD8^+^ T-cells in lung metastases of ccRCC was associated with shorter survival. Conversely, a high density of NKp46^+^ cells in lung metastases was associated with improved survival in ccRCC [12]. Additionally, brain metastases of ccRCC show the largest numbers of CD3+, CD8+ and PD1 positive cells compared with metastases from other primary tumors, but no correlation with survival was found [13]. Moreover, tumors with a higher Fuhrman grade had greater numbers of CD8^+^ lymphocytes in lung metastases, and a higher frequency of CD8+ lymphocytes in metastatic lesions was subsequently correlated with poor prognosis [14].

This study aims to examine the distribution of several immune cell subtypes in primary ccRCC and their distant metastases, at different sites, in the context of clinico-pathological data, and its correlation with survival.

## 2. Materials and Methods

### 2.1. Material

Fifty patients who underwent partial or radical nephrectomy for ccRCC between 2003–2014 at the Department of Urology, University Hospital Bonn were included in this study. Formalin-fixed, paraffin-embedded (FFPE) material from metastases resections obtained from 25 patients were also included in the study. Patient data are depicted in Table 1. The study was approved by the ethics committee of the University Hospital Bonn (EK 233/20).

For confirmatory studies, tissue microarrays (TMAs) of FFPE of 241 patients who underwent partial or radical nephrectomy for renal tumors at the Department of Urology, Charité Berlin (Berlin, Germany) were included in this study (Table 1). The study was approved by the ethics committee of the University Hospital Charité Berlin (EA1/134/212).

### 2.2. Methods

CD103/ITGAE expression in The Cancer Genome Atlas (TCGA)—Kidney Renal Clear Cell Carcinoma (KIRC) data set was used.

RNA-Seq by Expectation Maximization (RSEM)-normalized RNA-Seq data from TCGA, as well as follow-up data, were obtained from OncoLnc public database (www.oncolnc.org), accessed on 30 August 2021, as previously described [15]. Non-neoplastic tissue was excluded. CD103/ITGAE expression was dichotomized according to the median value of CD103/ITGAE and further analyses of clinico-pathological data and survival were performed. Additionally, hematoxylin and eosin (HE) slides from the TCGA-KIRC project (https://cancer.digitalslidearchive.org/#!/CDSA/kirc, accessed on 19 January 2022) were reevaluated for histological type and grading according to the International Society of Urological Pathology (ISUP). We excluded cases with ambiguous histology and cases with incomplete clinico-pathological or survival data. The analyzed data set comprises a total of 436 patients.

### 2.3. Immunohistochemistry

FFPE tissue sections were cut into 3 µm sections. Sections were stained with the following antibodies: CD3 (clone 565, NCL-L-CD3-565, dilution 1:50, Leica, Wetzlar, Germany), CD4 (clone SP35, 790-4425, ready-to-use-antibody, Roche, Basel, Switzerland), CD8 (clone C8/1448, M 7103, dilution 1:50, Agilent Technologies, Santa Clara, CA, USA), CD20 (clone L26, M 0755, dilution 1:2000, Agilent Technologies), using the Autostainer 480 S (Medac Diagnostika, Wedel, Germany) after boiling the slides at 99 °C for 20 min at pH 8.0. to pH 6.0. All supplementary reagents were purchased from Medac.

CD103 staining (clone EPR4, ab 129202, dilution 1:50, Abcam, Cambridge, UK) was performed using the Benchmark Ultra Autostainer (Ventana Medical Systems, Oro Valley, AZ, USA) after heat-induced epitope retrieval (HIER). HIER was performed by boiling the slides at 99 °C, for 20 min at pH 8.0. All supplementary reagents used for CD103 staining were purchased from Ventana Medical Systems.

All tissue sections were counterstained with Mayer′s hematoxylin (Merck, Darmstadt, Germany).

Positive controls were performed for each staining.

### 2.4. Quantification

Positive cells were counted on histology slides in five representative high-power fields in the tumor center, tumor periphery and metastases. In particular, for CD103, only highly stained cells were considered positive when background staining was high. Only small round cells with the typical lymphocyte morphology were counted. These values were then used to calculate the mean value. In metastases, positive cells in five representative high-power fields were counted and the mean value of positive cells was calculated. An immune score comprising the number of intratumoral CD3, CD4, CD8 and CD20 positive cell infiltrates was then calculated. Cases with an immune score above the mean were defined as having a high immune infiltration, whereas cases with low immune infiltration included those with a score below the mean. On TMAs, the percentages of CD8+ and CD103+ cells within each core were quantified by QuPath 2.3, and the mean value was calculated for each case.

### 2.5. Flow Cytometry Analysis

Fresh tumor material from three patients with ccRCC (RCC_016, RCC_018, RCC_026) was washed with ice-cold Advanced DMEM/F-12 (Gibco™, Thermo Fisher Scientific, Waltham, MA, USA) supplemented with Normocin (InvivoGen, San Diego CA, USA). Tumors were then dissociated into single cells using the Tumor Dissociation Kit, human, in gentleMACS C Tubes, and processed on a GentleMACS™ Octo Dissociator with Heaters (all from Miltenyi Biotec, Bergisch Gladbach, Germany) according to the manufacturer’s instructions. The resulting single-cell suspensions were washed once with Dulbecco’s Modified Eagle Medium (DMEM) (Gibco™, Thermo Fisher Scientific, Waltham MA, USA). Red blood cell (RBC) lysis was performed using Red Blood Cell Lysing Buffer Hybri-Max™ (Sigma-Aldrich, St. Louis, MI, USA) incubated at room temperature for 5 min. Cell suspensions were stained first with Human TruStain FcX (1/200 dilution) and Zombie NIR™ Fixable Viability Kit (1/400 dilution) (both from BioLegend, San Diego, CA, USA) diluted in DPBS (Gibco™, Thermo Fisher Scientific) for 10 min, at room temperature, in the dark. Staining of surface markers was then performed using the following antibodies: PerCP/Cyanine5.5 anti-human CD3 Antibody (UCHT1; 1 μg/mL), Brilliant Violet 711™ anti-human CD103 (Integrin αE) Antibody (Ber-ACT8; 1 μg/mL), APC/Cyanine7 anti-human CD4 Antibody (SK3; 0.12 μg/mL), and Spark Blue™ 550 anti-human CD8 Antibody (SK1; 2 μg/mL), diluted in DPBS. Samples were incubated for 15 min, at room temperature, in the dark. Samples were filtered through a 35 μm cell strainer prior to acquisition on a 4-laser Cytek Aurora (Cytek Biosciences, Fremont, CA, USA). All flow cytometry data were analysed on FlowJo (version 10.8.0; Becton Dickinson and company (BD), Franklin Lakes, NJ, USA) and graphs were plotted on Prism 9 (version 9.2.0; GraphPad, San Diego, CA, USA).

### 2.6. Statistics

Comparisons between variable clinical pathological groups were made using the Mann–Whitney test and the Kruskal–Wallis test.

For the FACS data, statistical significance was determined using a one-way analysis of variance (ANOVA) with Tukey’s multiple comparisons test.

Correlation analyses between variable groups were determined by Spearman’s rank correlation coefficient (for non-parametric data) and Pearson’s correlation coefficient (for parametric data). Survival analysis was performed using the log-rank test of Kaplan–Meier survival curves.

For the survival analysis, the best cut-off value was first determined as described by Budczies et al. [16] (Cut-off, Berlin cohort: CD103 1.267%, CD8 8.955%).

*p*-values under 0.05 were considered significant and *p*-values between 0.05–0.1 were considered to indicate a statistical trend. Statistical analyses were performed using IBM SPSS 25.0 unless specified otherwise.

## 3. Results

### 3.1. The Association of Clinico-Pathological Data with the Immune Cell Infiltrate

**Figure 1:**
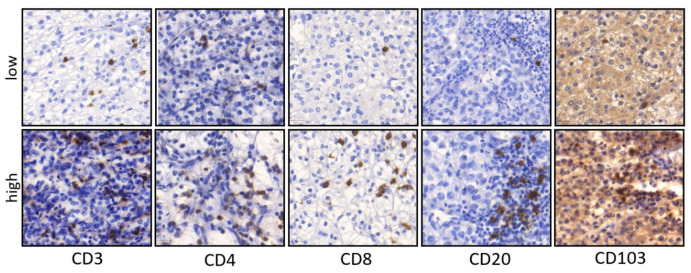
Representative pictures of IHC staining in primary ccRCC. Examples for low and high densities of CD3, CD4, CD8, CD20 and CD103 positive cells in primary ccRCC (×200 magnification).

To investigate the relationship between immune cell infiltration and clinico-pathological characteristics, immunohistochemistry staining was performed on ccRCC sections and the number of intratumoral and peritumoral CD3+, CD4+, CD8+, CD20+ and CD103+ lymphocytes was quantified (Figure 1). The mean value of immune cells was then correlated with the clinico-pathological data. Tumors with distant metastases had more intratumoral CD3+ lymphocytes than tumors without distant metastases (*p* = 0.032 Table 2). In the context of grading, better differentiated tumors (Grades 1 and 2) had significantly more peritumoral CD3+ lymphocytes than poorly differentiated tumors (Grades 3 and 4) (*p* = 0.011, Table 3).

No significant differences in the number of CD3+, CD4+, CD8+, and CD103+ lymphocytes were observed between the center and the periphery of ccRCC tumors or between organ-confined (pT1/2) and non-organ-confined (pT3/4) tumors.

No significant differences in the immune scores, calculated from the numbers of CD3, CD4, CD8 and CD20 cells, were found between organ-confined (pT1/2) and non-organ-confined (pT3/4) tumors, nor between well-differentiated (G1/G2) and poorly differentiated (G3/G4) tumors.

### 3.2. Comparison of Immune Cell Infiltration in Primary Tumors and Paired Distant Metastases

**Figure 2:**
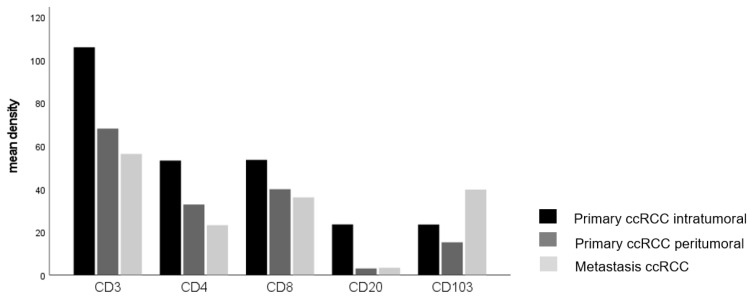
The main density of lymphocytes in ccRCC infiltrating the central and peripher tumor, and metastases (CD103 *p* = 0.032, Kruskal–Wallis-test).

Given that the amount of immune cell infiltrate differed in cases with and without distant metastases, we assessed the differences in immune cell infiltration between primary tumors and paired distant metastases. We found a significant accumulation of CD103+ cells in tumor metastases compared to their corresponding primary tumor (*p* = 0.032, Figure 2, Table 4), whereas the numbers of the other immune cells were significantly lower in tumor metastases compared to their primary tumor (Table 4).

Immune cell infiltration in distant metastases was composed of more CD3+, CD4+, CD8+, CD20+ and CD103+ lymphocytes in cases where the primary tumor was organ-confined compared with advanced, non-organ-confined tumors (Table 5), but only CD20+ lymphocyte infiltration showed statistical significance (*p* = 0.039).

### 3.3. Lung Metastases Have a Higher Immune Cell Infiltration than Other Localizations

**Figure 3:**
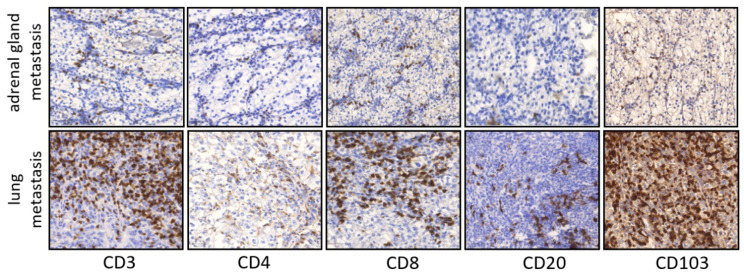
Representative pictures of IHC staining in primary ccRCC in metastatic lesions. Examples CD3, CD4, CD8, CD20 and CD103 infiltration in adrenal gland metastasis and lung metastasis (×100 magnification).

Our cohort comprises metastases across a number of sites including six lung metastases, four lymph node metastases, seven bone metastases and eight metastases from other sites (e.g., liver). Analyses of immune cell infiltration with respect to the localization of metastases revealed that lung metastases had a significantly higher infiltration of CD3+, CD4+ and CD103+ cells compared with other sites (*p* = 0.025, *p* = 0.006, *p* = 0.023, respectively, Table 6, Figure 3).

### 3.4. The Number of CD103+ Lymphocytes Correlates with the Infiltration of CD4+ and CD8+ T-Lymphocytes

Correlation analyses indicated a highly significant positive correlation between the densities of intratumoral CD8+ and CD103+ T-lymphocytes (r = 0.380; *p* = 0.013). Similarly, the densities of intratumoral CD4+ and CD103+ T-lymphocytes were also positively correlated (r = 0479; *p* = 0.002).

**Figure 4:**
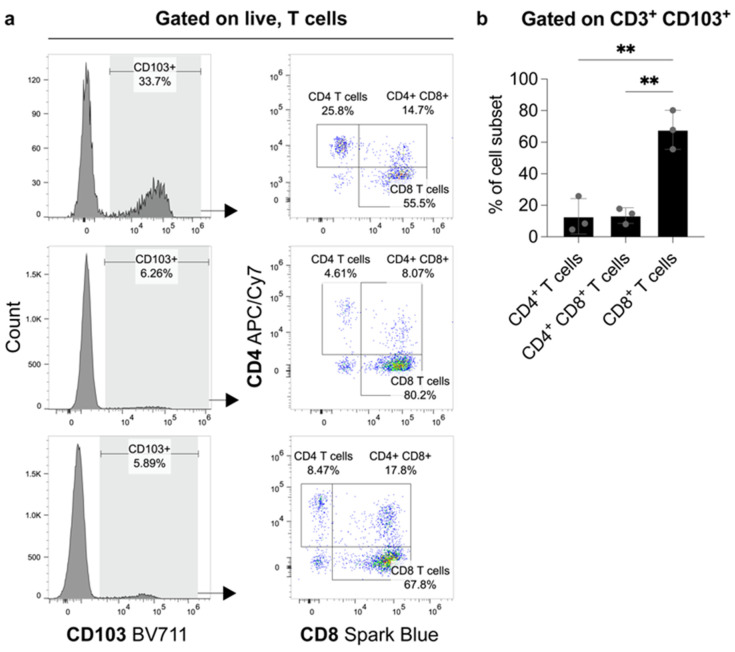
FACS analysis from fresh tumor material of three patients with ccRCC. The proportions of CD4+- and CD8+-expressing cells amongst CD103+ T-lymphocytes. (**a**) Flow cytometry plots show the frequency of CD103 expression on live, T-cells (gated on Zombie NIR- CD3+) and the subsequent distribution of CD4+ and CD8+ expressing cells amongst these CD103+ T-lymphocytes. The proportions of CD4+, CD4+ CD8+ and CD8+ cells as a frequency of CD3+ CD103+ cells are graphed in (**b**). N = 3 biological replicates. Error bars represent means ± standard deviation (SD) with each point representing one biological replicate. Statistical significance was determined using a one-way analysis of variance (ANOVA) with Tukey’s multiple comparisons test (** *p*-value < 0.01).

Flow cytometry analysis showed that CD4- CD8+ cells comprised the largest proportion of CD103+ CD3+ T-lymphocytes. In comparison, CD4+ CD8- cells and CD4+ CD8+ cells made up a significantly smaller portion of CD103+ CD3+ T-lymphocytes (Figure 4).

### 3.5. High Infiltration of CD103+ and CD8+ T-Lymphocytes Correlates with Poor Prognostic Parameters and Shorter Survival in ccRCC

Of the further 241 cases in the validation cohort (Table 7), CD103+ and CD8+ T-lymphocyte counts were available for 197 ccRCC cases. Poorly differentiated ccRCC (ISUP Grading 3 and 4) and cases with distant metastases showed significantly more intratumoral CD103+ lymphocytes than well-differentiated cases or ccRCC without distant metastases (*p* < 0.001 and *p* = 0.015, respectively). No differences in the numbers of CD103+ T-lymphocytes were observed when comparisons of tumor stages were made. Additionally, no significant differences in the numbers of CD8+ T-lymphocytes were found in relation to clinico-pathological parameters (Table 7).

**Figure 5:**
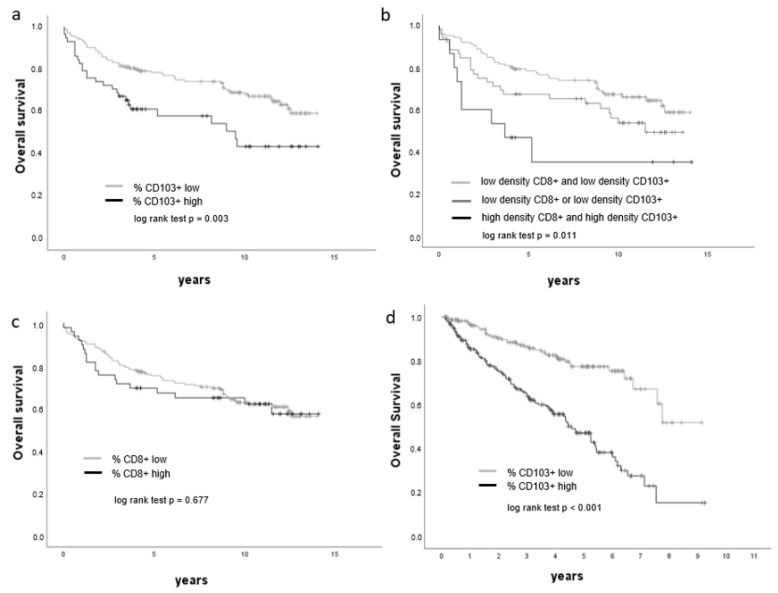
Overall survival is associated with CD103+ lymphocyte density. (**a**–**d**) Kaplan–Meier curves showing the overall survival of ccRCC patients with respect to cell densities of CD8+, CD103+ and CD103+ CD8+ cells, respectively (**a**–**c**: Berlin cohort, **d**: TCGA KIRC cohort).

A prognostic potential for CD103+ lymphocyte infiltration was indicated by Kaplan–Meier estimates. Specifically, a high density of CD103+ lymphocytes correlated with shorter overall survival (log-rank *p* = 0.014, Figure 5a). In a similar manner, a high density of CD103+ and CD8+ lymphocytes was predictive of poorer survival outcome (log-rank *p* = 0.013, Figure 5b). In contrast, the frequency of CD8+ T-lymphocytes alone was not a prognostic marker in ccRCC (Figure 5c). Furthermore, multivariate cox regression analysis using the percentage of CD103+ or CD8+ lymphocytes, T stage, grading and metastasis identified the density of CD103+ cells as an adverse prognostic marker (*p* = 0.009, Table 8).

### 3.6. mRNA Expression of ITGAE (CD103) Is Increased in ccRCC and Correlates with Poor Prognosis

Analysis of tumoral ITGAE (CD103) expression in a data set from TCGA indicated high expression (>median value) in 221 patients and low expression (<median value) in 215 patients. Further analyses showed that ITGAE (CD103) expression was significantly higher in non-organ-confined tumors compared to organ-confined tumors (pT3/4 versus pT1/2, *p* < 0.001), in poorly differentiated tumors compared with well-differentiated tumors (ISUP Grade 3/4 versus ISUP Grade 1/2, *p* < 0.001) and in distant metastases compared with cases without distant metastases (M1 versus M0, *p* < 0.001, Table 9). Additionally, ccRCC patients with high intratumoral ITGAE (CD103) expression were found to have significantly shorter overall survival than patients with low ITGAE (CD103) expression within the tumor (Figure 5d). Specifically, the median overall survival of patients with low intratumoral expression of ITGAE (CD103) was 1286 days, whilst the median overall survival of patients with high ITGAE (CD103) expression was 1011 days.

## 4. Discussion

Immune evasion, which permits tumor cells to escape immunological destruction, is a hallmark of cancer [17]. Many studies have observed that immune infiltrate in tumors can actively promote tumor growth and invasiveness [18,19]. The mechanisms of tumor progression by immune cells are complex and comprise a network of cytokine, chemokine and growth factors, as well as the production of reactive oxygen species and activation of signaling pathways [19].

Many studies focus on deciphering the role of immune cells in primary tumors; however, little is known about the role of immune cells in the distant metastases. Distant metastases, the primary targets of immune checkpoint blockades, often exhibit variable responses to immune therapy. Such variable responses could be due to differences in the immune contexture.

In this study, the comprehensive analysis of immune cell infiltration in ccRCC revealed that the densities of intratumoral and peritumoral lymphocytes vary with each case. We observed a high number of T-lymphocytes, identified by CD3 staining, in line with the data published by Chevrier et al. [20]. We subsequently analyzed the relationship between immune cell infiltration and the clinico-pathological characteristics of ccRCC. Whilst we found no association between immune cell infiltration and tumor stage, but tumor grading was observed to be inversely associated with T-lymphocytes (CD3+). Along these lines, a previous report by Davidsson et al., described an association between increased tumoral infiltration of CD4+ FoxP3+ lymphocytes with higher tumor grading, although no association with tumor stage was found [21]. The contradictory observations regarding tumor grading and the amount of CD3+ lymphocyte infiltration could be due to the small number (*n* = 50) of patients included in our study but also to the fact that the CD4+ FoxP3+ lymphocytes are only a subgroup of lymphocytes. Furthermore, a comparison of the immune cell infiltrate in tumors with and without metastases indicated that tumors with distant metastases had significantly more intratumoral CD3+ lymphocytes in the primary tumor than non-metastasized tumors, supporting a previous finding by Giraldo et al. [22] that the accumulation of T-lymphocytes in ccRCC is correlated with poor prognosis. In contrast to the CD3+ lymphocytes, CD4+ and CD8+ lymphocytes showed a tendency to be more frequent in M0 compared to M1. CD3 is a pan-marker for T-lymphocytes and is positive in all subtypes. This could also indicate that CD3+ cells contain other cell populations, such as γδ T-cells, which have shown promising results in cancer therapy [23].

The majority of studies investigating tumor-infiltrating lymphocytes (TIL) in cancer focus on T-lymphocyte subsets whose prognostic significance is broadly accepted. However, little is known about the role of B-lymphocytes in cancer progression or therapy response. A comprehensive meta-analysis of 69 published papers across several cancer subtypes revealed conflicting evidence statements pertaining to the prognostic value of B-lymphocytes [24]. In our study, the number of intratumoral B-lymphocytes in primary ccRCC, identified as CD20+ cells, did not show an association with tumor stage, grading or metastases. In distant metastasis, a high infiltration density was associated with organ-confined primary tumor stage. Published data on CD20+ cell tumor infiltration show different effects: Stenzl et al. [25] observed that high numbers of intratumoral CD20+ lymphocytes were an independent prognostic factor for longer disease-free and overall survival. In contrast, a study published by Sjöberg et al. [26] demonstrated that high intratumoral infiltration by CD20+ lymphocytes correlated with a shorter overall survival in patients with ccRCC. Given the scarcity of published data pertaining to the role of B-lymphocytes, further studies aiming to investigate the dynamics and role of B-lymphocytes in the context of cancer progression and response to immune checkpoint inhibitors will be invaluable.

The importance of the immune landscape in distant metastases and its influence on therapy response and survival has been reported for several tumor types [27,28,29]. Notably, the prognostic significance of tumor-infiltrating T-lymphocytes in primary and metastatic lesions of advanced stage ovarian cancer has been shown [29].

There are few investigations into CD103+ lymphocytes in ccRCC. However, CD103+ lymphocytes infiltrating distant metastases in ccRCC have not been characterized yet. Comparing the composition of the immune infiltrate in primary tumors and paired distant metastases, we found a significant accumulation of CD103+ lymphocytes in lung metastases but not in other metastatic sites. A comparison of the immune cell infiltrates of lung metastases with those in other metastatic sites, such as bone or adrenal glands, revealed a significantly higher numbers of CD3+, CD4+ and CD103+ cells. This suggests that the lung is a metastatic site in ccRCC with high frequencies of immune cell–tumor cell interactions. Regarding lung metastases, different results were described for colorectal carcinoma and hepatocellular carcinoma, in which CD3+ and CD8+ cells did not accumulate in lung metastases compared to primary tumors [30,31].

In two independent ccRCC cohorts, we observed an accumulation of CD103+ lymphocytes. An accumulation of CD103+ CD8+ positive lymphocytes in ccRCC has also been previously described by Dornieden et al. [32]. In addition to this population, the authors also described subpopulations of CD4+ CD103+ positive cells, as well as natural killer (NK) and mucosal-associated invariant T (MAIT)-cells with CD69 and CD103 co-expression, in renal tumors. In line with this, we sought to quantify the composition of CD3+ CD103+ cells in ccRCC by flow cytometry. Our analyses show that intratumoral CD3+ CD103+ cells were dominated by CD8-expressing lymphocytes and that CD4+ or CD4+ CD8+ lymphocytes made up a significantly smaller proportion of CD103+ T-lymphocytes. In contrast to these results showing that CD103+ cells are dominantly composed of CD8+ T-cells concerning grading, these cells show a different tendency: whereas CD103+ cells significantly accumulate in less differentiated tumors, CD8+ cells show a tendency to be more frequent in better differentiated tumors. This could result from the fact that CD103+ cells are only a subgroup of CD8+ cells.

In recent years, CD103 (integrin α_E_) has been described as a surface marker of tissue-resident memory cells. Whilst the role of tissue-resident memory cells is poorly understood, the accumulation of CD103+ lymphocytes has been associated with good prognosis in several cancer subtypes, including urothelial carcinoma and ovarian cancer [33,34]. In lung cancer, the accumulation of CD103+ lymphocytes was reportedly associated with survival benefit through improved T-cell receptor (TCR) antigen sensitivity, leading to rapid T-cell-mediated recognition and higher cytotoxicity against tumor cells [35].

In our study, survival analysis indicated that high numbers of tumor-infiltrating CD103+ cells were correlated with poor survival. Additionally, high *ITGAE (CD103)* expression in ccRCC patients correlated with significantly shorter overall survival compared to cases with low *ITGAE (CD103)* expression. Furthermore, multivariate analysis showed that the density of CD103+ cells in tumors was an independent, adverse prognostic factor for ccRCC patients. The findings in our study are contrary to the results reported by Zhou et al., showing that CD103+ lymphocytes in the peritumoral stroma in ccRCC were associated with improved prognosis [10]. These contradictory findings could be explained by differences in the cell types analyzed, the localization of the analyzed cells and differences in the antibodies used. Our results are further supported by analyses of mRNA expression from TCGA data and validated in two independent cohorts.

The association between CD103+ lymphocyte accumulation and poor prognosis described in our study could be related to an immunosuppressive and tolerogenic phenotype of CD103+ cells in ccRCC. Previously, a heterogeneous effect of CD8+ CD103+ tissue-resident memory-like cells was described in tuberculous pleural effusion, where they orchestrate immunosuppressive as well as immune-activating roles [36]. Given that the upregulation of CD103 on T-lymphocytes has been reported to be dependent on the stimulation of the TCR and on signaling through the TGF-β receptor [37,38], an impaired stimulation could suppress the antitumoral activity of CD103-expressing T-lymphocytes. In humans, TGF-β is produced by regulatory T-cells or by dendritic cells, both of which are often dysfunctional in ccRCC [39,40]. In primary lung cancer, tumor cells regulate CD8+ T-cell recruitment and induction of CD103 expression via expression of integrin α_v_ [41]. Further studies deciphering the mechanisms involved in CD103+ cell accumulation in ccRCC and lung metastases are necessary to reveal potential tumor progression and therapy resistance mechanisms.

## 5. Conclusions

Altogether, the results presented in this study indicate that CD103+ lymphocytes accumulate in lung metastases, demonstrate that a high CD103+ cell density in ccRCC is an adverse prognostic marker of survival and highlight the complex role of the tumor immune microenvironment. Considering the increased clinical application of immune checkpoint inhibitor therapies, our findings further highlight the need for studies that aim to elucidate the immune landscape of metastatic sites and its role in the development of therapy resistance.

## Figures and Tables

**Table 1:** Patient characteristics of both cohorts (Bonn and Berlin) and TCGA.

	Berlin	Bonn	TCGA KIRC
Sex			
Male	158 (65.6%)	30 (60%)	18 (46.2%)
Female	83 (34.4)	20 (40%)	21 (53.8%)
Missing	0	0	397
Age			
Mean	60.65	64.78	60.90
Range	30–86	39–79	26–90
pT stage			
pT1	134 (55.5%)	12 (24%)	227 (52.1%)
pT2	22 (9.1%)	8 (16%)	52 (11.9%)
pT3	82 (34.0%)	30 (60%)	150 (34.4%)
pT4	3 (1.2%)	0 (0%)	7 (1.5%)
pN stage			
pN0/cN0	223 (92.5%)	45 (90%)	Data missing
pN1	18 (7.5%)	5 (10%)	Data missing
Distal metastasis	31 (12.9%)	25 (50%)	73 (16.7%)
Grading			
G1	38 (16.4%)	1 (2%)	86 (19.7%)
G2	122 (52.6%)	32 (64%)	209 (47.9%)
G3	54 (23.3%)	13 (26%)	102 (23.4%)
G4	18 (7.8%)	4 (8%)	39 (8.9%)
Missing	9	0	0
Total	*n* = 241	*n* = 50	*n* = 436

**Table 2:** Correlation of intratumoral immune cell infiltrate with clinico-pathological parameters (Mann–Whitney U test, Bonn cohort, 50 patients).

Immune Cell Type Intratumoral		M0	M1	pT1/2	pT3/4	G1/G2	G3/G4
CD3	Mean	92.22	118.10	107.45	104.98	110.72	98.28
	*p*-value	0.032		0.408		0.399	
CD4	Mean	46.82	62.79	51.27	57.92	56.95	51.84
	*p*-value	0.124		0.867		0.588	
CD8	Mean	44.73	55.83	47.43	53.39	51.54	49.03
	*p*-value	0.286		0.827		0.868	
CD20	Mean	2.60	2.80	3.84	1.99	2.52	3.14
	*p*-value	0.219		0.547		0.937	
CD103	Mean	16.20	15.70	16.18	16.36	17.80	13.36
	*p*-value	0.062		0.699		0.90	

**Table 3:** Comparison of peritumoral immune cell infiltrate and clinico-pathological data (Mann–Whitney U test, Bonn cohort, *n* = 50 patients).

Immune Cell Type Peritumoral		M0	M1	pT1/2	pT3/4	G1/G2	G3/G4
CD3	Mean	92.51	117.73	107.42	104.90	110.66	98.22
	*p*-value	0.125		0.110		0.011	
CD4	Mean	48.00	61.90	51.28	57.91	56.94	51.86
	*p*-value	0.225		0.124		0.100	
CD8	Mean	55.00	60.90	47.43	55.43	53.78	49.03
	*p*-value	0.851		0.738		0.094	
CD20	Mean	26.00	28.20	23.71	30.04	30.16	22.36
	*p*-value	0.318		0.148		0.892	
CD103	Mean	23.70	24.90	22.63	25.72	29.33	15.51
	*p*-value	0.565		0.442		0.335	

**Table 4:** Comparison of the immune cell infiltration between primary tumors and paired metastases (Kruskal–Wallis test, Bonn cohort, *n* = 25 patients).

Immune Cell Type	Intratumoral Mean Value	Peritumoral Mean Value	Metastases Mean Value	*p*-Value
CD3	104.62	67.36	56.20	<0.001
CD4	53.94	31.94	23.13	<0.001
CD8	52.93	39.78	6.87	0.005
CD20	23.65	2.90	3.32	<0.001
CD103	2.84	2.70	9.50	0.032

**Table 5:** Comparison of immune cells infiltrating metastases of ccRCC in relation to clinico-pathological data (Mann–Whitney U test, Bonn cohort, *n* = 25 patients).

Immune Cell Type		pT1/2	pT3/4	G1/G2	G3/G4
CD3	Mean	82.00	38.86	65.50	34.57
	*p*-value	0.157		0.631	
CD4	Mean	29.55	17.86	23.133	26.23
	*p*-value	0.481		0.962	
CD8	Mean	39.88	11.23	34.05	38.17
	*p*-value	0.778		0.720	
CD20	Mean	5.15	4.4	3.67	2.16
	*p*-value	0.039		0.123	
CD103	Mean	52.90	20.58	43.15	24.23
	*p*-value	0.206		0.720	

**Table 6:** Comparison of the immune cell infiltration in distant metastases (Mann–Whitney U test, Bonn cohort, *n* = 25 patients).

Immune Cell Type	Lung Metastasis	Other Metastasis	*p*-Value
CD3 (mean)	150.93	27.37	0.025
CD4 (mean)	63.60	16.00	0.006
CD8 (mean)	49.47	24.33	0.412
CD20 (mean)	6.77	1.00	0.158
CD103 (mean)	69.80	16.17	0.023

**Table 7:** Comparison of intratumoral immune cells in relation to clinico-pathological data (Mann–Whitney U test, Berlin cohort, *n* = 197 patients).

Immune Cell Type		M0	M1	pT1/2	pT3/4	G1/G2	G3/G4
%CD8	Mean	6.11	10.27	6.90	5.60	6.53	1.07
	*p*-value	0.050		0.514		0.094	
%CD103	Mean	0.79	2.28	0.82	1.19	0.76	1.54
	*p*-value	0.015		0.199		<0.001	

**Table 8:** Multivariate Cox regression analyses (Berlin cohort, *n* = 197 patients, *p* < 0.001).

	HR (95% Confidence Interval)	*p*-Value
pT	1.529 (1.302–1.797)	<0.001
Grading	1.711 (1.109–2.639)	0.015
M	2.460 (1.399–4.325)	0.002
%CD103	1.146 (1.034–1.269)	0.009
%CD8	0.986 (0.957–1.015)	0.327

**Table 9:** TCGA CD103 Expression (Mann–Whitney U test, TCGA KIRK cohort, *n* = 436 patients).

		M0	M1	pT1/2	pT3/4	G1/G2	G3/G4
CD103	Mean	167.57	237.35	162.72	208.63	155.25	199.58
	*p*-value	<0.001		<0.001		<0.001	

## Data Availability

Not applicable.

## References

[1] Moch H., Cubilla A.L., Humphrey P.A., Reuter V.E., Ulbright T.M. (2016). The 2016 WHO Classification of Tumours of the Urinary System and Male Genital Organs-Part A: Renal, Penile, and Testicular Tumours. Eur. Urol..

[2] Fisher R., Gore M., Larkin J. (2013). Current and future systemic treatments for renal cell carcinoma. Semin. Cancer Biol..

[3] Grimm M.-O., Leucht K., Grünwald V., Foller S. (2020). New First Line Treatment Options of Clear Cell Renal Cell Cancer Patients with PD-1 or PD-L1 Immune-Checkpoint Inhibitor-Based Combination Therapies. J. Clin. Med..

[4] George D.J., Martini J.-F., Staehler M., Motzer R.J., Magheli A., Escudier B., Gerletti P., Li S., Casey M., Laguerre B. (2018). Immune Biomarkers Predictive for Disease-Free Survival with Adjuvant Sunitinib in High-Risk Locoregional Renal Cell Carcinoma: From Randomized Phase III S-TRAC Study. Clin. Cancer Res..

[5] Choueiri T.K., Figueroa D.J., Fay A.P., Signoretti S., Liu Y., Gagnon R., Deen K., Carpenter C., Benson P., Ho T.H. (2015). Correlation of PD-L1 tumor expression and treatment outcomes in patients with renal cell carcinoma receiving sunitinib or pazopanib: Results from COMPARZ, a randomized controlled trial. Clin. Cancer Res..

[6] Zhang C., Li Z., Qi F., Hu X., Luo J. (2019). Exploration of the relationships between tumor mutation burden with immune infiltrates in clear cell renal cell carcinoma. Ann. Transl. Med..

[7] Ghatalia P., Gordetsky J., Kuo F., Dulaimi E., Cai K.Q., Devarajan K., Bae S., Naik G., Chan T.A., Uzzo R. (2019). Prognostic impact of immune gene expression signature and tumor infiltrating immune cells in localized clear cell renal cell carcinoma. J. Immunother. Cancer.

[8] Duhen T., Duhen R., Montler R., Moses J., Moudgil T., de Miranda N.F., Goodall C.P., Blair T.C., Fox B.A., McDermott J.E. (2018). Co-expression of CD39 and CD103 identifies tumor-reactive CD8 T cells in human solid tumors. Nat. Commun..

[9] Djenidi F., Adam J., Goubar A., Durgeau A., Meurice G., de Montpréville V., Validire P., Besse B., Mami-Chouaib F. (2015). CD8+CD103+ tumor-infiltrating lymphocytes are tumor-specific tissue-resident memory T cells and a prognostic factor for survival in lung cancer patients. J. Immunol..

[10] Zhou J., Liu L., Yang T., Lu B. (2018). Prognostic and therapeutic value of CD103+ cells in renal cell carcinoma. Exp. Ther. Med..

[11] Wang L., Yang G., Zhao D., Wang J., Bai Y., Peng Q., Wang H., Fang R., Chen G., Wang Z. (2019). CD103-positive CSC exosome promotes EMT of clear cell renal cell carcinoma: Role of remote MiR-19b-3p. Mol. Cancer.

[12] Remark R., Alifano M., Cremer I., Lupo A., Dieu-Nosjean M.-C., Riquet M., Crozet L., Ouakrim H., Goc J., Cazes A. (2013). Characteristics and clinical impacts of the immune environments in colorectal and renal cell carcinoma lung metastases: Influence of tumor origin. Clin. Cancer Res..

[13] Harter P.N., Bernatz S., Scholz A., Zeiner P.S., Zinke J., Kiyose M., Blasel S., Beschorner R., Senft C., Bender B. (2015). Distribution and prognostic relevance of tumor-infiltrating lymphocytes (TILs) and PD-1/PD-L1 immune checkpoints in human brain metastases. Oncotarget.

[14] Bersanelli M., Gnetti L., Varotti E., Ampollini L., Carbognani P., Leonardi F., Rusca M., Campanini N., Ziglioli F., Dadomo C.I. (2019). Immune context characterization and heterogeneity in primary tumors and pulmonary metastases from renal cell carcinoma. Immunotherapy.

[15] Anaya J. (2016). OncoLnc: Linking TCGA survival data to mRNAs, miRNAs, and lncRNAs. PeerJ Comput. Sci..

[16] Budczies J., Klauschen F., Sinn B.V., Győrffy B., Schmitt W.D., Darb-Esfahani S., Denkert C. (2012). Cutoff Finder: A comprehensive and straightforward Web application enabling rapid biomarker cutoff optimization. PLoS ONE.

[17] Hanahan D., Weinberg R.A. (2011). Hallmarks of cancer: The next generation. Cell.

[18] DeNardo D.G., Andreu P., Coussens L.M. (2010). Interactions between lymphocytes and myeloid cells regulate pro- versus anti-tumor immunity. Cancer Metastasis Rev..

[19] Grivennikov S.I., Greten F.R., Karin M. (2010). Immunity, inflammation, and cancer. Cell.

[20] Chevrier S., Levine J.H., Zanotelli V.R.T., Silina K., Schulz D., Bacac M., Ries C.H., Ailles L., Jewett M.A.S., Moch H. (2017). An Immune Atlas of Clear Cell Renal Cell Carcinoma. Cell.

[21] Davidsson S., Fiorentino M., Giunchi F., Eriksson M., Erlandsson A., Sundqvist P., Carlsson J. (2020). Infiltration of M2 Macrophages and Regulatory T Cells Plays a Role in Recurrence of Renal Cell Carcinoma. Eur. Urol. Open Sci..

[22] Giraldo N.A., Becht E., Pagès F., Skliris G., Verkarre V., Vano Y., Mejean A., Saint-Aubert N., Lacroix L., Natario I. (2015). Orchestration and Prognostic Significance of Immune Checkpoints in the Microenvironment of Primary and Metastatic Renal Cell Cancer. Clin. Cancer Res..

[23] Yazdanifar M., Barbarito G., Bertaina A., Airoldi I. (2020). γδ T Cells: The Ideal Tool for Cancer Immunotherapy. Cells.

[24] Wouters M.C.A., Nelson B.H. (2018). Prognostic Significance of Tumor-Infiltrating B Cells and Plasma Cells in Human Cancer. Clin. Cancer Res..

[25] Stenzel P.J., Schindeldecker M., Tagscherer K.E., Foersch S., Herpel E., Hohenfellner M., Hatiboglu G., Alt J., Thomas C., Haferkamp A. (2020). Prognostic and Predictive Value of Tumor-infiltrating Leukocytes and of Immune Checkpoint Molecules PD1 and PDL1 in Clear Cell Renal Cell Carcinoma. Transl. Oncol..

[26] Sjöberg E., Frödin M., Lövrot J., Mezheyeuski A., Johansson M., Harmenberg U., Egevad L., Sandström P., Östman A. (2018). A minority-group of renal cell cancer patients with high infiltration of CD20+B-cells is associated with poor prognosis. Br. J. Cancer.

[27] Mlecnik B., van den Eynde M., Bindea G., Church S.E., Vasaturo A., Fredriksen T., Lafontaine L., Haicheur N., Marliot F., Debetancourt D. (2018). Comprehensive Intrametastatic Immune Quantification and Major Impact of Immunoscore on Survival. J. Natl. Cancer Inst..

[28] Halama N., Michel S., Kloor M., Zoernig I., Benner A., Spille A., Pommerencke T., von Knebel D.M., Folprecht G., Luber B. (2011). Localization and density of immune cells in the invasive margin of human colorectal cancer liver metastases are prognostic for response to chemotherapy. Cancer Res..

[29] Leffers N., Gooden M.J.M., de Jong R.A., Hoogeboom B.-N., ten Hoor K.A., Hollema H., Boezen H.M., van der Zee A.G.J., Daemen T., Nijman H.W. (2009). Prognostic significance of tumor-infiltrating T-lymphocytes in primary and metastatic lesions of advanced stage ovarian cancer. Cancer Immunol. Immunother..

[30] Ahtiainen M., Elomaa H., Väyrynen J.P., Wirta E.-V., Kuopio T., Helminen O., Seppälä T.T., Kellokumpu I., Mecklin J.-P. (2021). Immune Contexture of MMR-Proficient Primary Colorectal Cancer and Matched Liver and Lung Metastases. Cancers.

[31] Woo H.Y., Rhee H., Yoo J.E., Kim S.H., Choi G.H., Kim D.Y., Woo H.G., Lee H.S., Park Y.N. (2022). Lung and lymph node metastases from hepatocellular carcinoma: Comparison of pathological aspects. Liver Int..

[32] Dornieden T., Sattler A., Pascual-Reguant A., Ruhm A.H., Thiel L.G., Bergmann Y.S., Thole L.M.L., Köhler R., Kühl A.A., Hauser A.E. (2021). Signatures and Specificity of Tissue-Resident Lymphocytes Identified in Human Renal Peritumor and Tumor Tissue. J. Am. Soc. Nephrol..

[33] Wang B., Wu S., Zeng H., Liu Z., Dong W., He W., Chen X., Dong X., Zheng L., Lin T. (2015). CD103+ Tumor Infiltrating Lymphocytes Predict a Favorable Prognosis in Urothelial Cell Carcinoma of the Bladder. J. Urol..

[34] Webb J.R., Milne K., Watson P., deLeeuw R.J., Nelson B.H. (2014). Tumor-infiltrating lymphocytes expressing the tissue resident memory marker CD103 are associated with increased survival in high-grade serous ovarian cancer. Clin. Cancer Res..

[35] Abd Hamid M., Colin-York H., Khalid-Alham N., Browne M., Cerundolo L., Chen J.-L., Yao X., Rosendo-Machado S., Waugh C., Maldonado-Perez D. (2020). Self-Maintaining CD103+ Cancer-Specific T Cells Are Highly Energetic with Rapid Cytotoxic and Effector Responses. Cancer Immunol. Res..

[36] Yu S., Lao S., Yang B., Wu C. (2021). Tissue-Resident Memory-Like CD8+ T Cells Exhibit Heterogeneous Characteristics in Tuberculous Pleural Effusion. J. Immunol. Res..

[37] Ling K.-L., Dulphy N., Bahl P., Salio M., Maskell K., Piris J., Warren B.F., George B.D., Mortensen N.J., Cerundolo V. (2007). Modulation of CD103 expression on human colon carcinoma-specific CTL. J. Immunol..

[38] Nizard M., Roussel H., Diniz M.O., Karaki S., Tran T., Voron T., Dransart E., Sandoval F., Riquet M., Rance B. (2017). Induction of resident memory T cells enhances the efficacy of cancer vaccine. Nat. Commun..

[39] Herber D.L., Cao W., Nefedova Y., Novitskiy S.V., Nagaraj S., Tyurin V.A., Corzo A., Cho H.-I., Celis E., Lennox B. (2010). Lipid accumulation and dendritic cell dysfunction in cancer. Nat. Med..

[40] Gigante M., Blasi A., Loverre A., Mancini V., Battaglia M., Selvaggi F.P., Maiorano E., Napoli A., Castellano G., Storkus W.J. (2009). Dysfunctional DC subsets in RCC patients: Ex vivo correction to yield an effective anti-cancer vaccine. Mol. Immunol..

[41] Malenica I., Adam J., Corgnac S., Mezquita L., Auclin E., Damei I., Grynszpan L., Gros G., de Montpréville V., Planchard D. (2021). Integrin-αV-mediated activation of TGF-β regulates anti-tumour CD8 T cell immunity and response to PD-1 blockade. Nat. Commun..

